# Smart Sensing Strip Using Monolithically Integrated Flexible Flow Sensor for Noninvasively Monitoring Respiratory Flow

**DOI:** 10.3390/s151229881

**Published:** 2015-12-15

**Authors:** Peng Jiang, Shuai Zhao, Rong Zhu

**Affiliations:** State Key Laboratory of Precision Measurement Technology and Instrument, Department of Precision Instrument, Tsinghua University, Beijing 100084, China; jiang-p13@mails.tsinghua.edu.cn (P.J.); zolmes2010@163.com (S.Z.)

**Keywords:** respiratory disease, wireless monitoring, micro sensor, flexible hot-film flow sensor, wearable device

## Abstract

This paper presents a smart sensing strip for noninvasively monitoring respiratory flow in real time. The monitoring system comprises a monolithically-integrated flexible hot-film flow sensor adhered on a molded flexible silicone case, where a miniaturized conditioning circuit with a Bluetooth4.0 LE module are packaged, and a personal mobile device that wirelessly acquires respiratory data transmitted from the flow sensor, executes extraction of vital signs, and performs medical diagnosis. The system serves as a wearable device to monitor comprehensive respiratory flow while avoiding use of uncomfortable nasal cannula. The respiratory sensor is a flexible flow sensor monolithically integrating four elements of a Wheatstone bridge on single chip, including a hot-film resistor, a temperature-compensating resistor, and two balancing resistors. The monitor takes merits of small size, light weight, easy operation, and low power consumption. Experiments were conducted to verify the feasibility and effectiveness of monitoring and diagnosing respiratory diseases using the proposed system.

## 1. Introduction

According to the report of World Health Organization (WHO), respiratory diseases have become one of the top major killers in the past decade [1]. Apnea Syndrome, Asthma, and Chronic Obstructive Pulmonary Disease (COPD) are the most common respiratory diseases that bring serious risks to human health, and even death. Sleep apnea is an involuntary cessation of breathing that occurs while the patient is asleep [2]. Obstructive sleep apnea syndrome (OSAS) directly relates to human respiration, which is suffered by 20%–40% of elderly people, and more than half patients remain undiagnosed [3]. Risk factors include being male, overweight, and over the age of 40. As a matter of fact, however, sleep apnea may strike anyone at any age, even children [4]. Most of respiratory diseases are chronic in nature and have a major impact not only on the individuals with diseases, but also on community, which bring about significant public health burdens. Therefore, effective methods of early detection and diagnosis are significantly necessary [5]. Respiratory airflow monitoring is vital to diagnose OSAS and other respiration diseases.

In order to diagnose OSAS, a system called polysomnography (PSG) [6] is commonly used in sleep labs and operated by physicians. PSG is the current preferred diagnostic modality but is relatively inconvenient, expensive, and has low efficiency [7]. Consequently, a portable monitor (PM) has been proposed as a substitute for PSG in pre-diagnostic assessment of patients with suspected OSA [8]. PM requires less technical expertise, is less labor intensive and time consuming, and is easier for patients to access [9,10]. Our group previously reported a wireless portable system for monitoring respiratory diseases. We used a micro thermal flow sensor to monitor respiratory airflow, a tri-axis micro accelerometer to monitor body posture, and a microphotoelectric sensor to monitor blood oxygen saturation [11].

Respiratory symptoms to be monitored including, but not limited to, effort, airflow, snoring, end-tidal CO_2_, esophageal pressure, breathing humidity. There are several kinds of respiratory sensors that have been reported. One is indirectly monitoring respiration from variation in thorax volume, the other is using airflow sensor to directly detect respiratory airflow [12]. The former method includes transthoracic impedance sensors, micro and macro bending fiber-optic sensors [13], 3D image sensors, and ultrasonic sensors, which could detect the variation of chest or abdominal circumference. However the indirect methods are sensitive to body movements, which may result in invalid measurements. Respiratory airflow is a key vital signal for monitoring respiratory disorder. The apnea and hypopnea index are more relevant with diagnosis of OSAS. Several kinds of airflow sensors are used to monitor respiratory airflow, such as pressure sensor, hot-wire/hot-film sensor, infrared thermography [14], and ultrasonic flowmeter [15]. The differential pressure flow sensor and the hot-wire/hot-film flow sensor are the most common flow sensor used in the portable medical instruments and some wearable devices. Differential pressure flow sensors generally need a pipeline and diaphragm structure. In recent years, both Fleisch pneumotahographs and variable orifice meters have a linear response; hence, they overcome the hurdle related to the low sensitivity at low flow rate of the fixed orifice meters [16,17]. The intensity-modulated fiber-optic sensors can monitor the humidity of exhaled airflow. The condensed humidity substantially alters the interaction between the light from the optical fiber and the surrounding medium, which can be monitored by a photo detector [13]. The IRT method can remotely sense the breathing dynamics by detecting the temperature variation of the exhale and inhale air [14]. Comparatively, micro hot-wire/hot-film sensors have a small size, simple structure, low cost, high sensitivity, and capability of integrating with on-chip circuitry [18]. For monitoring respiratory airflow, most monitors use nasal cannulas which have two prominent small pipes inserted into the nostrils to conduct nasal airflow to the flow sensors. The narrow tube may hinder nasal airflow and increase the respiratory resistance, thereby results in unsmooth and uncomfortable breathing for the patient. Furthermore, the long nasal cannula is easily squeezed by the human body and twined with limbs. Effective respiratory airflow monitor with noninvasive and comfortable detection is increasingly imperative.

In this paper, we propose a miniaturized flexible respiration monitor (termed smart strip) that can be attached on human upper lip underneath the nostril to implement non-invasive, wireless, and real-time respiratory flow monitoring without use of nasal cannula and mask. The device utilizes a tailor-designed micro flow sensor, which is monolithically-integrated with a temperature-compensating resistor and two balancing resistors on single chip. The monitoring system comprises a flexible flow sensor attached on a molded flexible silicone case, a miniaturized conditioning circuit with a low power Bluetooth4.0 LE module packaged in the case, and a personal mobile device that wirelessly acquires sensor data, executes extraction of vital signs, and performs medical diagnosis.

## 2. System Design

### 2.1. System Overview

**Figure 1:**
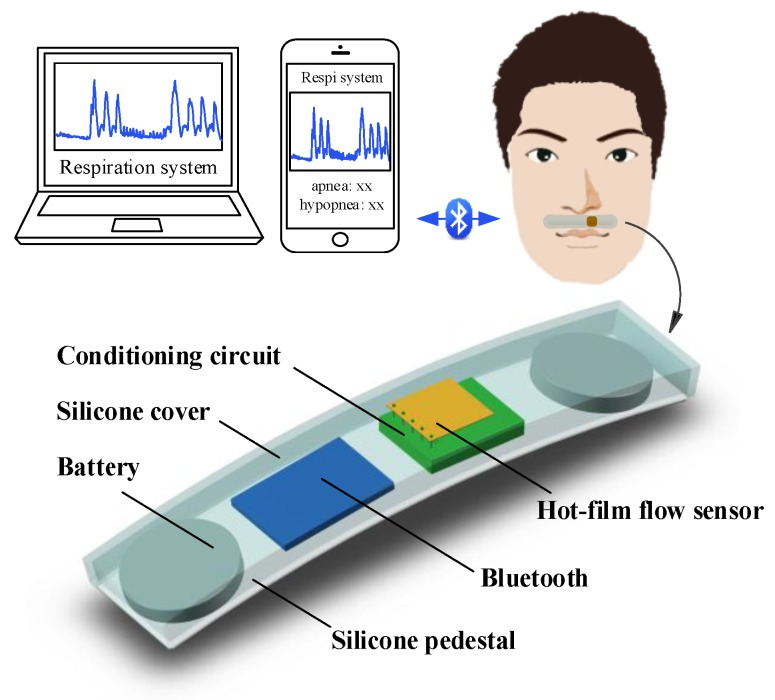
Schematic of the system.

**Figure 2:**
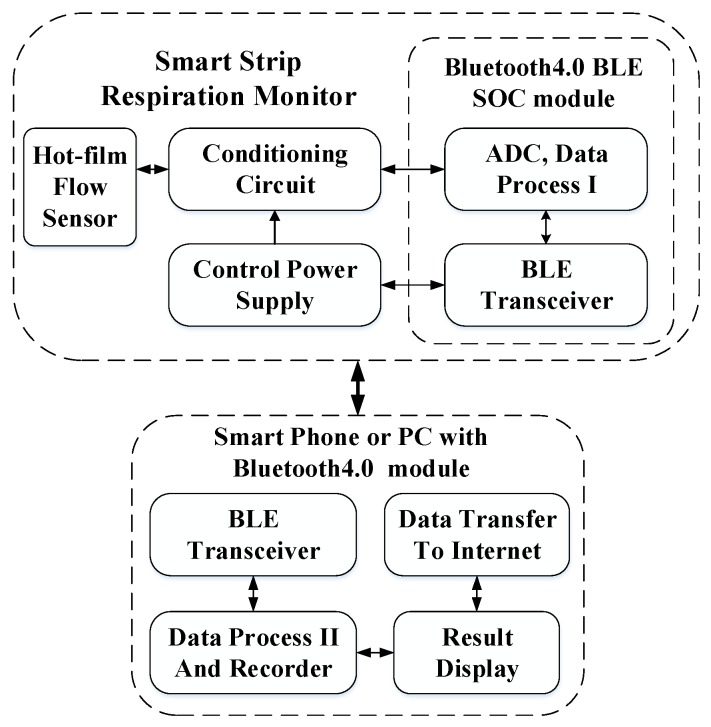
The block diagram of signal flow.

Figure 1 shows a flexible respiration monitor structured like a strip, which is comprised of a micro hot-film flow sensor, a signal conditioning circuit, a Bluetooth4.0 LE module, and rechargeable battery. The monitor is wirelessly connected with a personal computer (PC) or a smart phone via Bluetooth communication. The monitor (also termed smart strip of respiration monitor, SSRM) is flexible that can be attached on the upper lip underneath the nostrils. The respiratory flow breathed out from the nostrils blows over the strip and is detected in real-time by the hot-film flow sensor attached on the strip. Tube-free configuration makes the respiration monitoring non-invasive and unobstructed to the breath of the patient. Figure 2 shows the signal flow of the system. In the SSRM, the hot-film flow sensor is used to detect the respiratory flow, and the conditioning circuit acquires the sensor data. The Bluetooth4.0 LE module containing a 12 bit ADC, a MCU part, a GPIO part, and Bluetooth4.0 LE transceiver is used to submit respiration data to a smart phone or a PC. Smart phone or PC receives the respiration data, implements medical analysis, records, and displays the results.

### 2.2. Design and Fabrication of Monolithically Integrated Flexible Hot-Film Flow Sensor

Hot-film anemometers have been widely used in industry, automobile, medical, aerospace, and other fields. The micro-machined thermal flow sensor based on silicon technology was firstly developed in 1974 [19]. With the development of flexible electronics, several flexible flow sensors have been reported in recent years [20]. Petropoulos A. *et al.* developed a flexible gas flow sensor which was fabricated on a flexible PCB substrate and was able to quantify 0–10 SLPM flow rate [21]. Sturm H. *et al.*, developed a thermoelectric mass flow-rate sensor on a 10 μm thick polyimide foil [22]. Bowman B. *et al.*, developed a respiration sensor containing thermoresistive elements, which were screened on the substrate using conductive ink [23].

**Figure 3:**
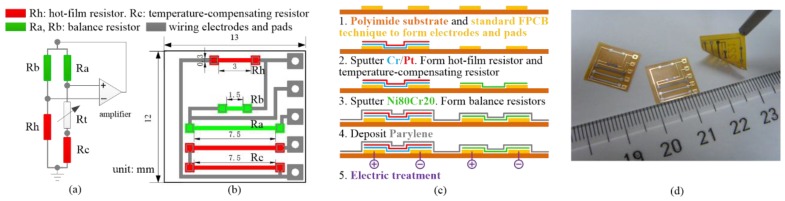
(**a**) CTD circuit of the flow sensor; (**b**) the design of the monolithically integrated hot-film flow sensor; (**c**) the fabrication processes of the sensor; and (**d**) fabricated sensors.

The integrated hot-film sensor is shown in Figure 3, combines four resistors on one chip, including a hot-film resistor Rh, a temperature compensating resistor Rc, and two balancing resistors Ra and Rb, all of which serve as legs of a Wheatstone bridge [24]. The fabrication of the sensor is shown in Figure 3c. The sensor is fabricated on a flexible polyimide substrate by incorporating the printed circuit technique with the micromachining sputter technique. The hot-film flow resistors and temperature-compensating resistors are made of platinum due to its high temperature coefficient of resistance (TCR). The two balancing resistors as fixed resistors are made of Nichrome (80/20 wt%), namely Ni80Cr20, due to its low thermal sensitivity and low conductivity. Figure 3d demonstrates the prototype of the fabricated sensor. The average TCR of the hot-film flow resistor and the temperature-compensating resistor is about 1400 ppm/K and the average TCR of two balancing resistors is around 70 ppm/K.

Though many micro hot-film flow sensors on silicon or polyimide substrate have been realized [19], monolithic integration of the flow sensor with its conditioning circuit, especially on a flexible substrate is still problematic. As mentioned above, to directly detect the respiratory airflow, an effective airflow sensor is important. In traditional methods, the airflow sensors are usually embedded into a tube to monitor the respiratory flow blowing through it. The respiratory flow is collected and pipelined by a nasal tube or nasal mask, which unavoidably hinders breathing. To overcome the problem, the proposed monitor adopts a monolithically integrated flexible hot-film flow sensor attached on a flexible silicone case which can be used to monitor comprehensive respiratory airflow in a noninvasive way.

The flow sensor utilizes the hot-film resistor Rh as both Joule heater and temperature detector. Under a constant bias power and zero flow rate, the hot-film resistor is heating and achieves a steady-state temperature, which means the heat transfer system reaches equilibrium. If an external airflow passes, the hot-film resistor experiences forced convective cooling. Accordingly, the temperature of the hot-film resistor decreases and makes the resistance changes, and thus provides the information of the flow rate that dominates the cooling rate. A constant temperature difference (CTD) mode [25] shown in Figure 3a is used to operate the hot-film sensor due to its advantages of high sensitivity and fast response, where Rh is the hot-film resistor with about 90 Ω at 23 °C, Rc is the temperature-compensating resistor with about 474 Ω, Rb and Ra are the balance resisters with 50 Ω and 263 Ω, Rt is an adjust resistor for modulating the overheat ratio of 0.5%.

**Figure 4:**
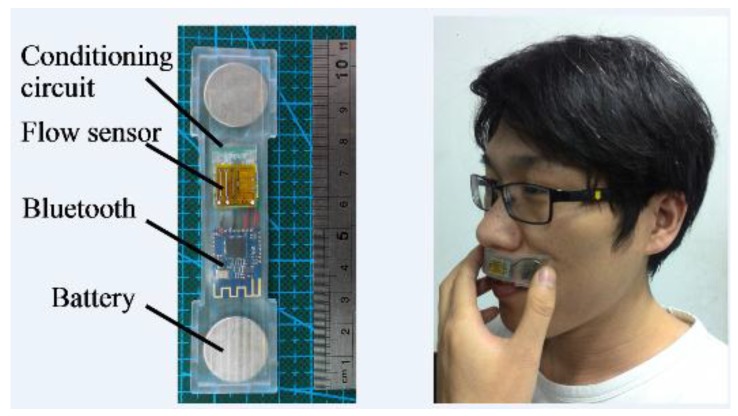
The prototype of developed monitor and demonstration of attaching it on the upper lip.

To make the monitor flexible and bendable so as to be capable to attach on the human upper lip, the flexible hot-film flow sensor was adhered on the outer surface of a molded flexible case. The packaging case of the monitor was molded by using polydimethylsiloxane (PDMS), which is flexible and biocompatible. The conditioning circuit of the hot-film sensor, the Bluetooth module, and the battery were distributed and packaged in the flexible case. Figure 4 shows a prototype of the developed SSRM with a size of 5 mm × 25 mm × 100 mm.

### 2.3. Low Power Consumption Design

**Figure 5:**
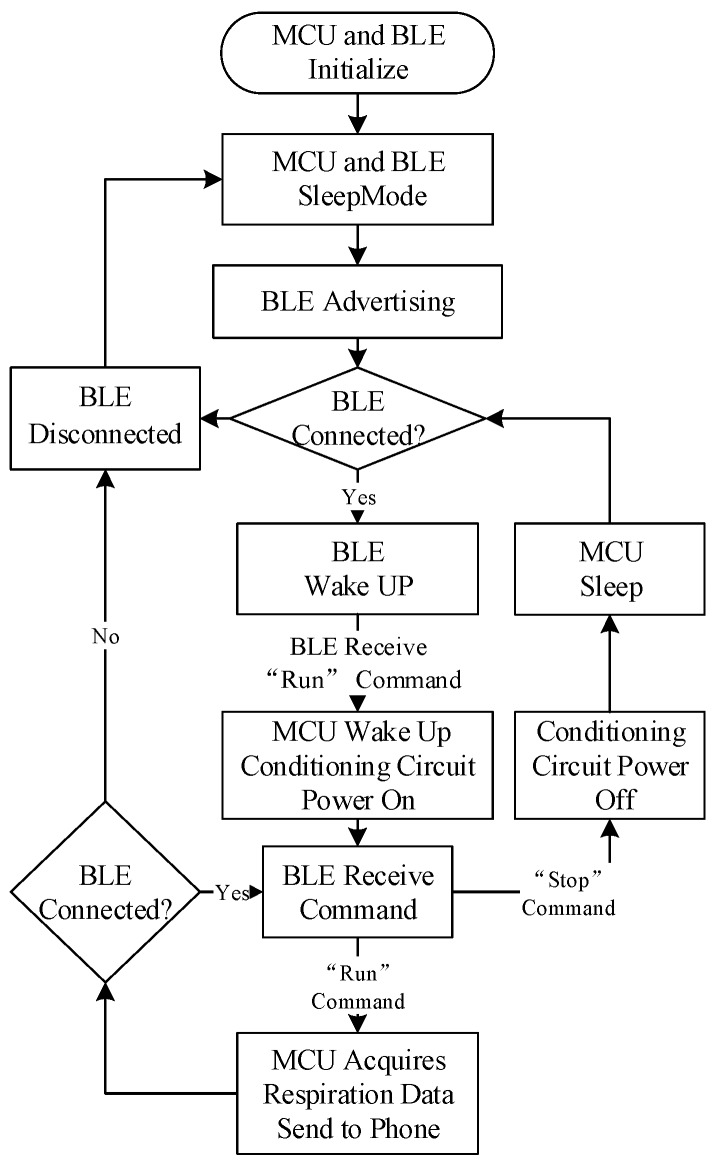
A flow chart of the SSRM.

Power consumption is an important issue to be considered in system design. Low-power components were selected, such as a low-power operational amplifier which has a power consumption of less than 100 μA at 3.3 V. The power consumption of the Wheatstone bridge was reduced by setting a low ratio of Rb/Rh (around 0.5) and using an appropriate overheat ratio (0.5%) to make a tradeoff between the sensitivity and the power consumption for the sensor. The average working power of the conditioning circuit of the hot-film sensor reached about 33 mW and the monitoring system consumption was 43 mW. To further save the energy, a sleep-mode circuit was used. When MCU received a “run” command, it opened the MOSFET to power the conditional circuit. If the MCU received a “stop” command, it shut down the MOSFET to make the conditioning circuit dormancy, and then the MCU entered into sleep mode. Bluetooth Low Energy (BLE) is an emerging low-power wireless technology developed for short-range communication and monitoring applications that is expected to be incorporated into billions of devices in the next few years [26]. In order to minimize power consumption, our monitor combined a Bluetooth4.0 LE module (DA14580) with an MCU to construct a digital data acquisition system. The average working power of the BLE module is only about 5 mW [27]. Use the MCU to control the conditioning circuit power supply and acquire the respiration data. Figure 5 shows a flow chart of the monitor. All techniques mentioned above saved power as much as possible to extend the battery life. In addition, a wireless battery charging chip with a low power consumption of 2 μA at 4.2 V was integrated within the SSRM to realize a wireless charging for the device.

### 2.4. Algorithm for Extracting Respiratory Parameters

An algorithm of extracting the respiratory parameters for analyzing and diagnosing respiratory diseases was designed. Minute ventilation (MV), peak inspiratory flow (PIF), respiratory rate (RR), and tidal volume (TV) are the key parameters of respiration. Respiratory minute volume (or minute ventilation or expired minute volume) is the volume of gas inhaled (inhaled minute volume) or exhaled (exhaled minute volume) from a person’s lung per minute [28]. Digital integration of acquired respiratory flow value in one minute gets MV, and the maximum value of respiratory peak in one minute is defined as PIF. Counting the peaks of respiration waveform in one minute gets the value of RR. Dividing the minute ventilation by the respiratory rate obtains the value of TV. Table 1 shows the respiratory parameters calculated from an experimental data where a health adult was tested in normal breathing. Table 1 also list typical normal human respiratory parameters as comparison.

**Table 1:** Tested respiratory parameters.

Parameters	MV (L)	TV (L)	PIF (L/min)	RR (min^−1^)
Tested Value	6.1	0.3	21	20
Normal Value	6–8	0.3–0.8	N/A	12–24

## 3. Experimental Results and Discussions

### 3.1. Simulation of Human Exhalation and Inhalation Flow Field

**Figure 6:**
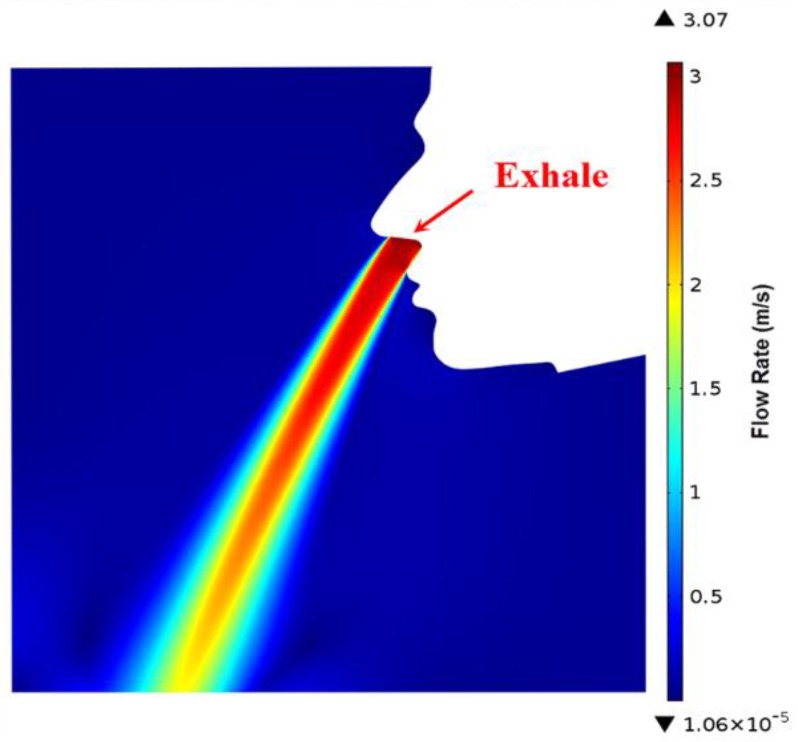
Simulation of human exhalation.

**Figure 7:**
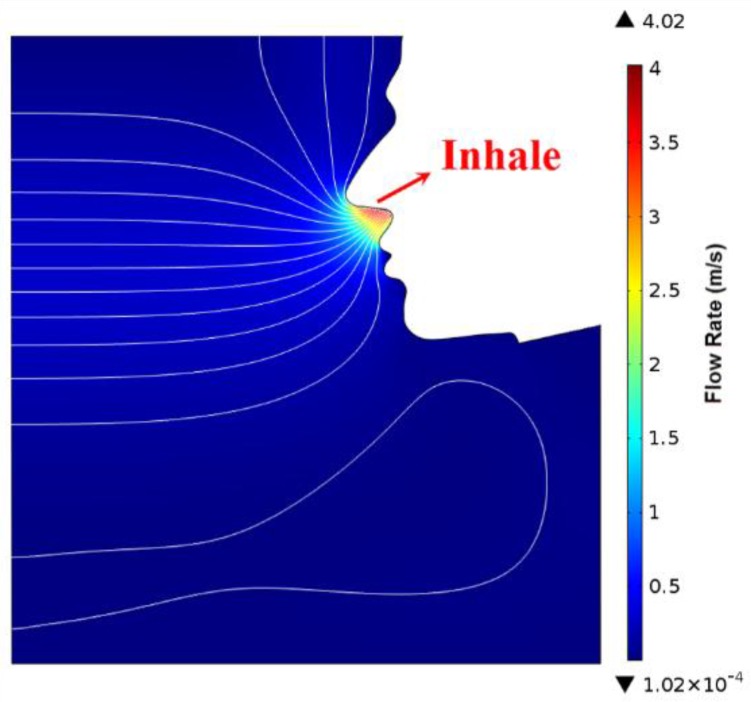
Simulation of human inhalation.

In order to theoretically verify the feasibility of the respiratory monitor, a computational fluid dynamic (CFD) simulation of human respiration airflow was conducted to demonstrate the ventilation and the flow distribution. We chose a k-ε RANS turbulent model [29] to calculate the flow field. The simulated exhalation and inhalation processes are shown in Figure 6 and Figure 7 respectively. In the exhalation process, the inlet was inside the nostril through which an expiration flow rate of 3 m/s blew out. The flow velocity of 3 m/s was determined according to a typical respiration flow rate 18 L/min. The diameter of the nostril was set as 8 mm corresponding to the anthropometric size of an adult. In the inhalation process, the pressure of the nostril as an outlet was set to be a pressure subtracting 20 Pa from a standard atmospheric pressure to simulate a normal inhale state. The negative pressure of 20 Pa was estimated according to the inhale flow rate. In both of exhalation and inhalation, the respiratory flow blew through the upper lip, was detected by the SSRM attached there.

### 3.2. Calibration and Dynamic Response of the Airflow Sensor

**Figure 8:**
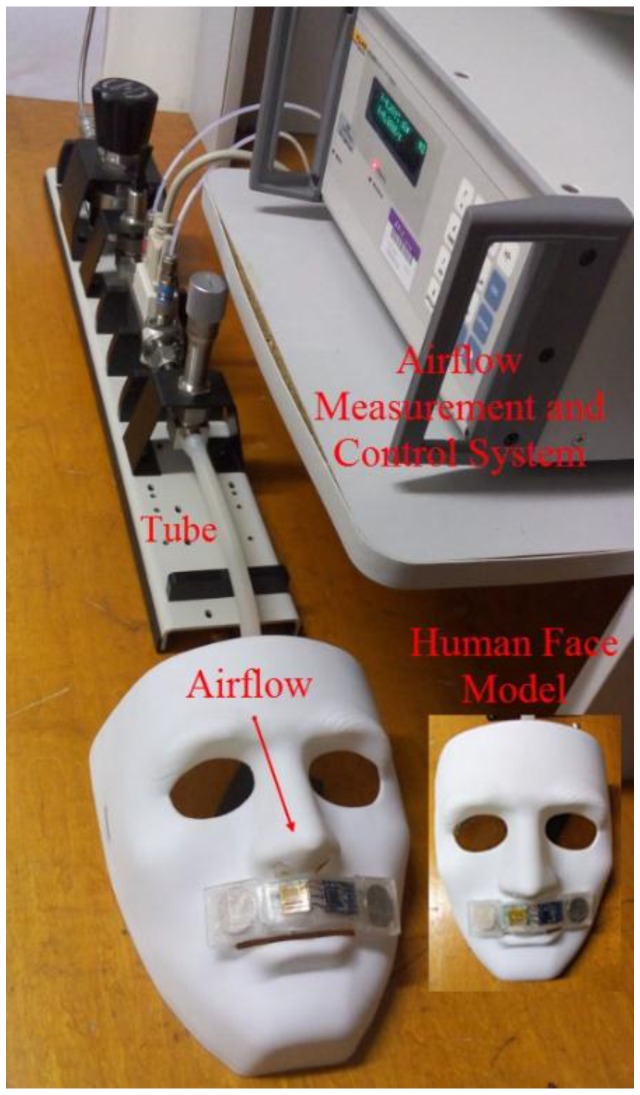
Experimental setup for airflow sensor calibration.

**Figure 9:**
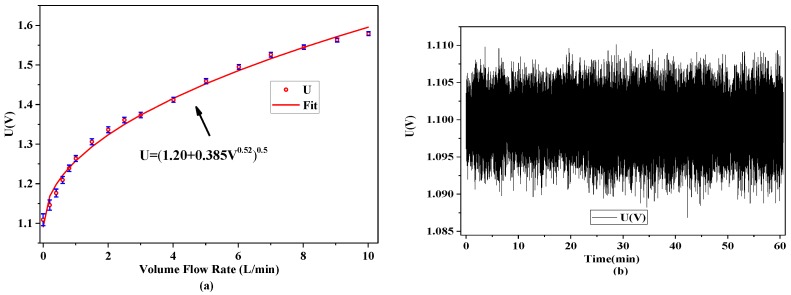
(**a**) Calibration of airflow sensor; and (**b**) stability of the sensor.

A calibration experiment was conducted to obtain the relationship between the respiratory flow and the output signal of the sensor (*i.e.*, the top voltage of the Wheatstone bridge). The experimental setup for calibration is shown in Figure 8. The developed SSRM was attached on the upper lip of human face model. The outlet of a pipe with an inner diameter of 9 mm was connected into the nostril of a face model, by which a controlled airflow was breathed out the nostril and blew through the SSRM. The inlet of the tube was connected to an airflow measurement and control system (Fluke MOLBOX1+) that accurately adjusted the airflow rate. Figure 9a illustrates the relationship between the airflow and the output voltage of the sensor. The uncertainty of the sensor outputs is estimated to be about ±5% and the bias stability of the sensor is about 5 mV as shown in Figure 9b. According to the King’s law, the relationship between the airflow (denoted as V) and the sensor output voltage (denoted as U) can be formulated as U^2^ = a + bV^n^, where a, b, and n are constants that were determined through the least squares estimation.

**Figure 10:**
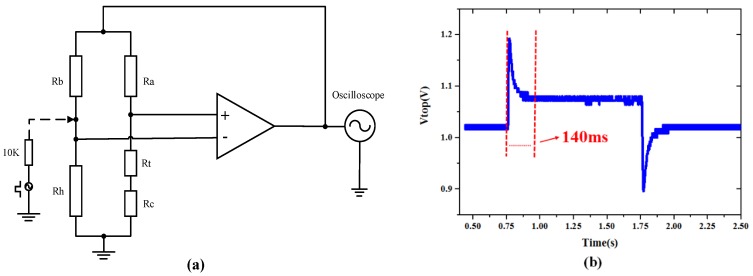
(**a**) Schematic conditioning circuit of sensor system; and (**b**) tested dynamic response.

The dynamic response of the hot-film sensor was tested to verify dynamically monitoring the respiratory airflow. An electrical excitation method was applied to measure the response time of the sensor. A schematic view of the conditioning circuit is shown in Figure 10a. A source of 10 mV square pulse voltage in serials with a limiting resistor (10 KΩ) generated a step excitation to the sensor. The dynamic response of the sensor was tested and shown in Figure 10b. The experimental result indicates the response time of the sensor is about 140 ms, which is consistent with the bandwidth of 7.2 Hz. A low-pass filter combined with an amplifier provided a 3 dB bandwidth of about 7.2 Hz. This bandwidth can fulfill the typical respiratory rate of an adult which is 12–20 bpm [30]. The sample rate of the monitoring system was set as 100 Hz. More experiments demonstrated that the flow range of the developed hot-film sensor covered 0–60 L/min, the temperature drift was less than ±3% in the ambient temperature variation from 25 °C to 50 °C [24]. The benefits of using this integrated micro hot-film flow sensor lie in: flexibility, easy fabrication and packaging, high sensitivity, capability to detect comprehensive respiratory parameters, and being extremely sensitive to weak flow. The flexible structure makes the sensor capable to be attached on curved surfaces.

### 3.3. Monitoring of Respiration

**Figure 11:**
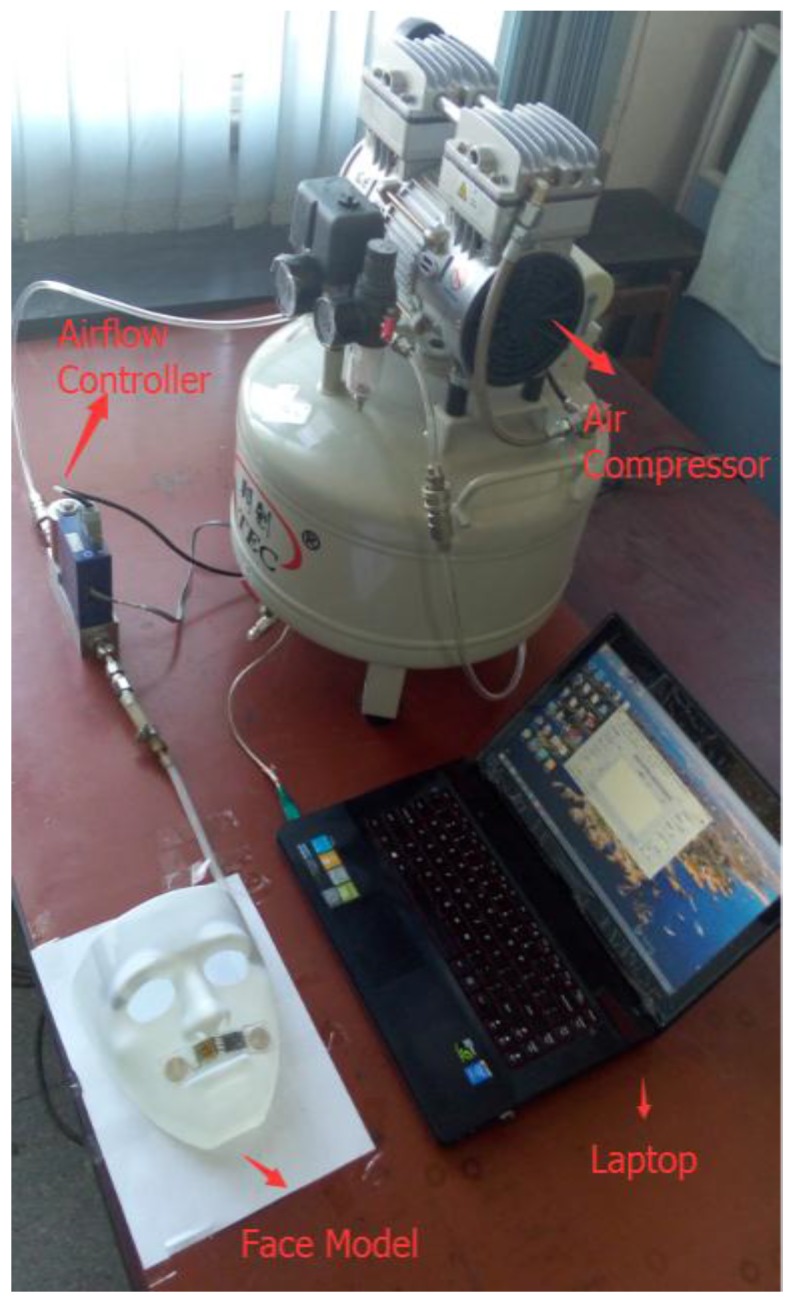
Experimental setup for respiration simulation.

**Figure 12:**
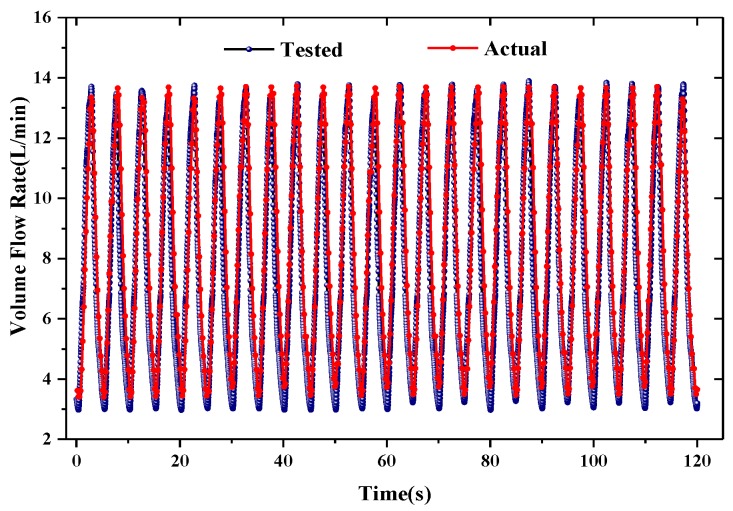
Simulation respiration signal.

An experiment of respiration simulation was conducted. Figure 11 shows the experimental setup for respiration simulation. An air compressor (FI-Tech AT80/38) and an airflow controller (Sevenstar CS230A) were used as a respiration generator. A respiratory rate of 12 bpm with a peak-to-peak airflow from 3.5 L/min to 13.9 L/min was generated and blew over the upper lip of the face model. The airflow was controlled and recorded by a PC, and the airflow signals detected by the SSRM were transmitted wirelessly to the PC as well. Figure 12 demonstrates a comparison of tested airflow and actual flow. It is seen that the airflow detected by the SSRM agreed very well with the actual airflow. Table 2 lists the respiratory parameters calculated from tested data by using the algorithm described in Section 2.

**Table 2:** Test results of respiratory parameters.

Parameters	MV (L)	TV (L)	PIF (L/min)	RR (min^−1^)
**Tested value**	8.0	0.67	14.2	12
**Actual value**	8.3	0.69	13.9	12

**Figure 13:**
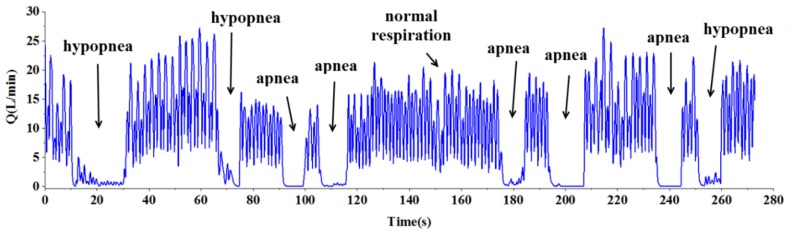
Apnea and hypopnea in real-time respiration signal.

To demonstrate diagnosing respiratory diseases, a sleep apnea recognition experiment was conducted. The experiments on human subjects were approved by Ethical Review Board of Tsinghua University (No. 20140084). The experiment for recognizing apnea and hypopnea was conducted in the way that at room temperature the subject wore the SSRM and breathed with simulated apneas and hypopneas while the respiratory flow was recorded. The proposed algorithm was applied to recognize apnea and hypopnea from the respiratory flow data detected in real-time. Experimental results are shown in Figure 13, where the apnea and hypopnea were recognized and the numbers of apnea and hypopnea episodes (AHI) were counted automatically. AHI is an index used to indicate the severity of sleep apnea, which is represented by the number of apnea and hypopnea occurring per hour in sleep (normal 0–5, mild 5–15, moderate 15–30, severe >30).

### 3.4. Monitoring of Oxygen Saturation and Respiration

**Figure 14:**
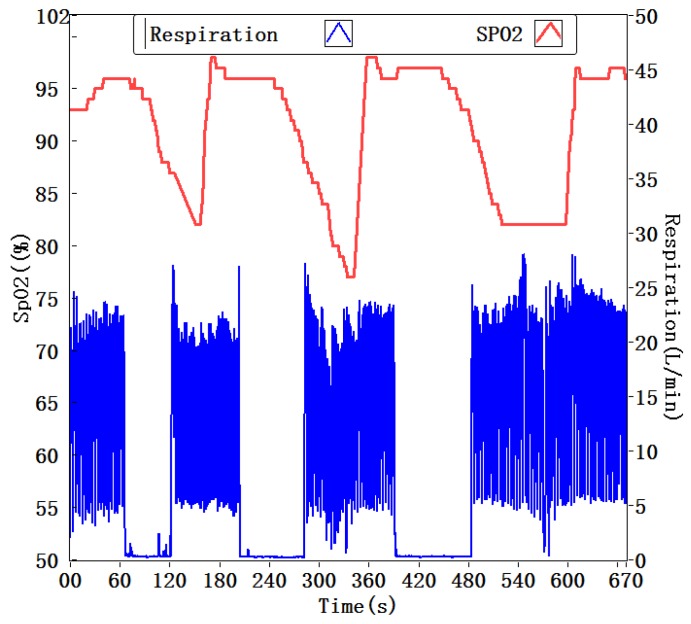
Monitoring of SpO_2_ and respiration.

Portable Monitoring Task Force of American Academy of Sleep Medicine (AASM) has made a recommendation: a portable OSA monitor uses oximetry as one of the monitoring channels [10,31]. When an OSA event occurs, the patient cannot inhale oxygen for a period of time, therefore the arterial oxygen saturation (SpO_2_) will gradually reduce. This is a distinct sign for identifying the apnea or hypopnea event. Combination of oximetry and SSRM to simultaneously monitor SpO_2_ and respiration enables more precise diagnosis for OSAS. An experiment was conducted by using a wrist pulse oximeter (CONTEC, CMS50F) worn on the subject’s finger and a SSRM attached on their upper lip. During the experiment, the subject artificially held their breath to simulate apnea three times. Each time the breath was held for about one minute. Figure 14 shows the tested results of the desaturation and resaturation of SpO_2_, as well as the inhale and exhale waveform of the respiration. The results indicate the index of SpO_2_ went down after an apnea occurring and exhibited a time delay. These experimental results were repeatable, and the correction between the incidences of apnea and SpO_2_ decreasing is higher than 95%. As mentioned above, no-breath or low-breath will result in SpO_2_ decreased. Normal index of SpO_2_ ranges from 95% to 100%. It was reported that oximetry analyzed by counting the number of arterial oxygen desaturations index for 4% concluded the OSA, but the diagnosis sensitivity was 40% and specificity was 98%. Serious OSAS patients suffer frequent occurrences of apneas accompanied by oxygen desaturation during sleep. A combination of respiratory and SpO_2_ monitoring could improve the accuracy of diagnosis for OSAS.

### 3.5. Monitoring of Sleep Posture, Oxygen Saturation, and Respiration

**Figure 15:**
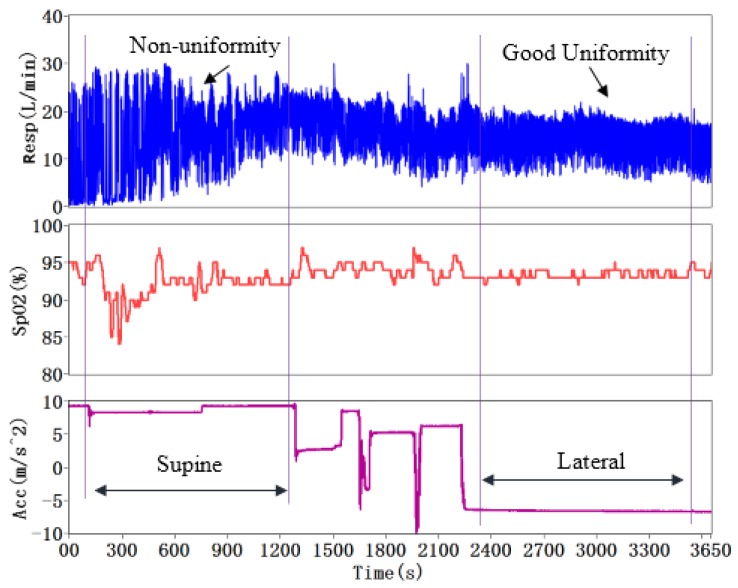
Monitoring of sleep posture, SpO_2_, and respiration.

Many medical studies have demonstrated that the sleep posture has a significant influence on the apneic events, and lateral decubitus can reduce the frequency of obstructive respiratory events [31]. The OSA patients at supine posture encounter with apnea/hypopnea events more than twice than those at lateral posture. The effect of gravity on the upper airway (UA) of the patient at the supine posture is the most dominant factor for the anatomical and physiological changes [32]. We conducted an experiment to demonstrate the relationship between body posture and human respiration. The SSRM, a homemade body posture sensor (using tri-axis accelerometer to identify the posture [13]) and a CMS50F pulse oximetry were wore on the subject and monitored continuously in sleep. Figure 15 shows the tested results of body posture, SpO_2_ and respiration. The body posture sensor was placed in front of the chest and the Z-axis of sensor was perpendicular to the body. When the subject was at supine posture, the magnitude of the Z-axis acceleration was around 1 g (namely 9.8 m/s^2^), otherwise at lateral posture. From the results, we can see the respiration exhibited non-uniformity at the supine posture. Several apneas occurred in the supine process. In contrast, the respiration at the lateral posture exhibited good uniformity. No apnea/hypopnea event occurred in this process. The experimental results demonstrated that the lateral posture can prevent apnea/hypopnea happening.

## 4. Conclusions

A wireless, non-invasive sensing system for monitoring real-time respiratory flow using a monolithically-integrated flexible hot-film flow sensor incorporated with a smart phone or PC is presented in the paper. The monitoring unit, like a smart strip, is flexible that can be attached on the human upper lip. The smart strip is comprised of flexible integrated hot-film flow sensor, conditioning circuit, rechargeable battery, and Bluetooth4.0 LE module, all of which are packaged in a flexible silicon case. The integrated hot-film flow sensor combines four resistors of a Wheatstone bridge operated in CTD mode on one chip, including a hot-film resistor, a temperature compensating resistor and two balancing resistors. As a wearable device, the tube-free configuration makes the respiratory monitoring non-invasive and minimal resistance and, therefore, the users could take a more comfortable experience. Low power consumption design is conducted via software and hardware optimization to meet the requirements for long-time monitoring, covering at least a sleep cycle of eight hours. This non-invasive respiratory monitor makes comprehensive respiration parameters acquirable, which are important in diagnosis of OSA, COPD, and asthma. In addition of respiration monitoring, the proposed devices can be also applied in the monitoring of newborns during nutritive suction [33].
